# Analysis of the Influence of Different Bionic Structures on the Noise Reduction Performance of the Centrifugal Pump

**DOI:** 10.3390/s21030886

**Published:** 2021-01-28

**Authors:** Cui Dai, Chao Guo, Yiping Chen, Liang Dong, Houlin Liu

**Affiliations:** 1 School of Energy and Power Engineering, Jiangsu University, Zhenjiang 212013, China; daicui@ujs.edu.cn (C.D.); 2211911008@stmail.ujs.edu.cn (Y.C.); 2 Research Center of Fluid Machinery Engineering and Technology, Jiangsu University, Zhenjiang 212013, China; 2221911012@stmail.ujs.edu.cn (C.G.); liuhoulin@ujs.edu.cn (H.L.)

**Keywords:** bionic blade, pit structure, sawtooth structure, noise reduction, centrifugal pump

## Abstract

The strong noise generated during the operation of the centrifugal pump harms the pump group and people. In order to decrease the noise of the centrifugal pump, a specific speed of 117.3 of the centrifugal pump is chosen as a research object. The bionic modification of centrifugal pump blades is carried out to explore the influence of different bionic structures on the noise reduction performance of centrifugal pumps. The internal flow field and internal sound field of bionic blades are studied by numerical calculation and test methods. The test is carried out on a closed pump test platform which includes external characteristics and a flow noise test system. The effects of two different bionic structures on the external characteristics, acoustic amplitude–frequency characteristics and flow field structure of a centrifugal pump, are analyzed. The results show that the pit structure has little influence on the external characteristic parameters, while the sawtooth structure has a relatively great influence. The noise reduction effect of the pit structure is aimed at the wide-band noise, while the sawtooth structure is aimed at the discrete noise of the blade-passing frequency (BPF) and its frequency doubling. The noise reduction ability of the sawtooth structure is not suitable for high-frequency bands.

## 1. Introduction

The mechanical noise and flow noise generated during the operation of the centrifugal pump are high, which not only affects the safe and reliable operation of the pump, but also endangers the physical and mental health of staff. The flow noise spreads to the far end along the pipeline and expands the harm range. Therefore, low-noise centrifugal pumps are being paid more and more attention [1,2,3].

Methods of improving the running state and reducing the noise of centrifugal pumps have been put forward by many researchers. Regarding reducing flow-induced noise by improving the geometric parameters of centrifugal pumps, the dynamic and static interference is the focus of research [4]. Tourret et al. [5] studied the noise of centrifugal pumps through tests, and pointed out that the area of most intense pressure fluctuation was located in the volute tongue. Dong et al. [6,7] studied the influence of different characteristics of the volute on the flow field. The results of this study stated that the increasing gap, stiffness and damping of the volute and impeller effectively reduce the noise of the centrifugal pump. Li et al. [8,9] studied the influence of a stepped volute tongue on the noise reduction performance of centrifugal pumps. The study shows that the gas flow near the stepped volute tongue is obviously improved compared with the traditional tongue, and the eddy current is reduced, thus reducing the noise. Velarde et al. [10] used numerical simulation and experimental methods to study the influence of impeller–volute interaction on the noise of a centrifugal fan, and analyzed the influence of the distance between impeller and tongue on impeller–volute interaction. As the core working component of the centrifugal pump, the geometric parameters of the impeller are very important for the flow-induced noise in the centrifugal pump. The noise reduction of impeller modification focuses on geometric shape, geometric parameters, and so on. Wu et al. [11,12] discussed the influence of inlet diameter, blade inlet angle, outlet width, and other impeller parameters on centrifugal pump noise. Tan [13,14] studied the influence of the unequal spacing of blades and the number of blades on the noise of the centrifugal pump. Dong et al. [6,7] studied the relationship between the size of the impeller and the noise, and reduced noise by adding short blades. Embleton [15] achieved the goal of reducing the noise of centrifugal fans by slotting the blades. Cravero et al. [16] carried out the unsteady simulation of the centrifugal blower and analyzed the influence of the main geometric parameters on the noise reduction effect. The effects of the main geometric design parameters on hydrodynamic and acoustic performance were determined.

With the development of bionics theory, researchers have studied the bionic noise reduction of fluid machinery by extracting unique noise reduction feature structures from biology. Yang et al. [17] arranged a pit structure on the surface of a car rear view mirror, and carried out a numerical simulation on the aerodynamic noise of the model. The results show that the pressure distribution and velocity distribution of the flow field were improved by the pit structure, and a hemispherical pit has the best noise reduction effect, reaching a noise reduction of 3–6 dB in the frequency region above 1000 Hz. Ge [18] employed the large eddy simulation numerical method to explore the internal flow field and acoustic performance of the bionic pit centrifugal pump, and verified that the pit structure has noise reduction properties in the centrifugal pump. Howe [19,20] first carried out a theoretical study on the noise reduction mechanism of a sawtooth trailing edge structure on a semi-infinite plate, and pointed out that the sawtooth trailing edge constituted an inclined trailing edge structure, making the noise radiation efficiency lower than the general structure. Ryi et al. [21] studied the noise reduction effect of a conventional sawtooth trailing edge and inclined trailing edge on a wind turbine rotor through tests. The results show that the noise reduction of a conventional sawtooth trailing edge is 0.59 dB higher than that of an inclined sawtooth trailing edge. Liu et al. [22] studied the noise reduction effect of a sawtooth trailing edge on a centrifugal fan through numerical simulation, and obtained a noise reduction effect of 9.8 dB. They pointed out that a sawtooth trailing edge reduces the disturbance of the falling vortex to wake and pressure fluctuation on the blade surface, which therefore reduces the aerodynamic noise caused by the trailing vortex. Gruber et al. [23,24,25,26,27] studied the noise reduction mechanism of a sawtooth trailing edge structure of different sizes through tests. They found that the sawtooth trailing edge structure reduces the trailing edge noise in the low-frequency range, but increases the trailing edge noise in the high-frequency range. Dai Cui et al. [28,29] studied the influence of groove structure on the noise reduction performance of centrifugal pumps, and explored the arrangement position of non-smooth structures on centrifugal pumps.

In summary, it can be found that the noise reduction effect is limited by adjusting the geometric parameters of volute and impeller. The technology of noise reduction using bionic non-smooth surfaces has gradually matured, but the research and applications in the field of centrifugal pumps are not extensive. Therefore, it is necessary to study the noise reduction effect of bionic structures in the field of centrifugal pumps and the influence of bionic structures on the flow and sound field of centrifugal pumps. In this paper, a high-precision numerical calculation of centrifugal pumps with different bionic structures is carried out to explore the influence of different bionic structures on noise reduction performance, so as to provide a powerful reference for the optimal design and performance improvement of the centrifugal pump.

## 2. Numerical Calculation

### 2.1. Model Parameters

**Figure 1:**
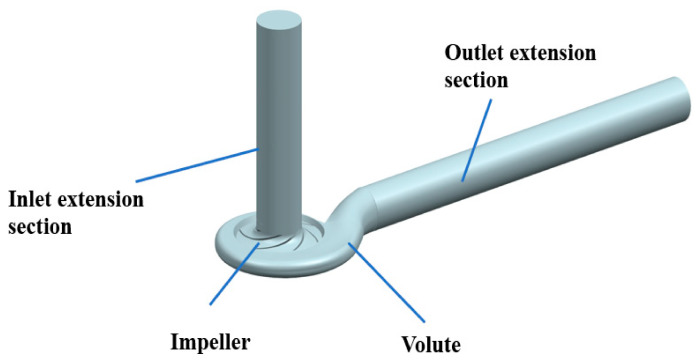
Three-dimensional model of calculated domain.

A specific speed 117.3 of the centrifugal pump is chosen as the research object. The main design parameters are flow rate *Q_d_* = 40 m^3^/h, head *H* = 8 m, and rotating speed *n* = 1450 r/min. Table 1 shows the main geometric parameters of the impeller and volute. The calculated domain mainly includes impeller, volute, and inlet and outlet extension section. The length of the inlet and outlet extension section is 8 times that of the inlet and the outlet diameter, respectively. The calculated domain is shown in Figure 1.

### 2.2. Bionic Structure Parameters

**Figure 2:**
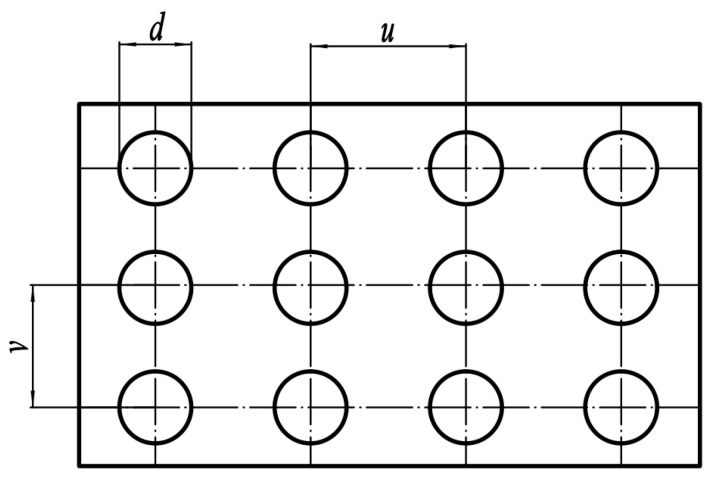
Rectangular arrangement of pit structure.

In order to compare the effects of different bionic structures on the noise reduction performance of the centrifugal pump, pit and sawtooth structures are arranged on the blade, respectively. The flow situation in the impeller near the pressure face is disordered, so the pits are arranged in the 1/3 nearby area of the pressure face outlet. The pit is arranged in a rectangle, as shown in Figure 2. In order to realize the scale change of the bionic pit structure along the flow direction, the most direct method is to make the value of each column different. According to the previous research of Chen [30], the pits are divided into three different groups in the flow direction, *d*_1_ = 1.79 mm, *d*_2_ = 2.07 mm, *d*_3_ = 1.42 mm, *u* = 1.75 mm, *v* = 1.75 mm.

**Figure 3:**
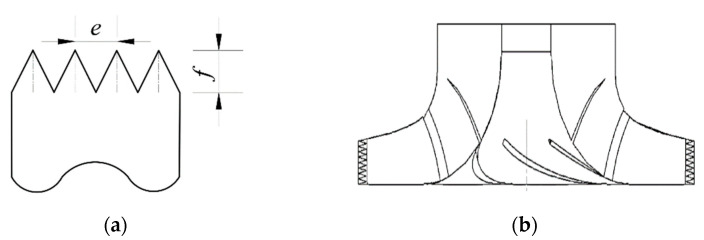
(**a**) Parameters of bionic sawtooth structure; (**b**) calculated domain of bionic sawtooth impeller.

In research of the noise reduction of bionic sawtooth structures, the dimensionless ratio of tooth distance *e* to tooth height *f* is generally taken as the key parameter. According to the study of Tong et al. [31], *e/f* = 0.4 is selected. The schematic diagram of the structure is shown in Figure 3a. Modeling methods of impeller sawtooth structures are mainly divided into embedding and cutting. Chong et al. [32,33,34,35,36,37] believed that the noise reduction effect of embedding is not as good as cutting. Therefore, cutting is adopted, and its calculated domain is shown in Figure 3b.

### 2.3. Grid Generation

**Figure 4:**
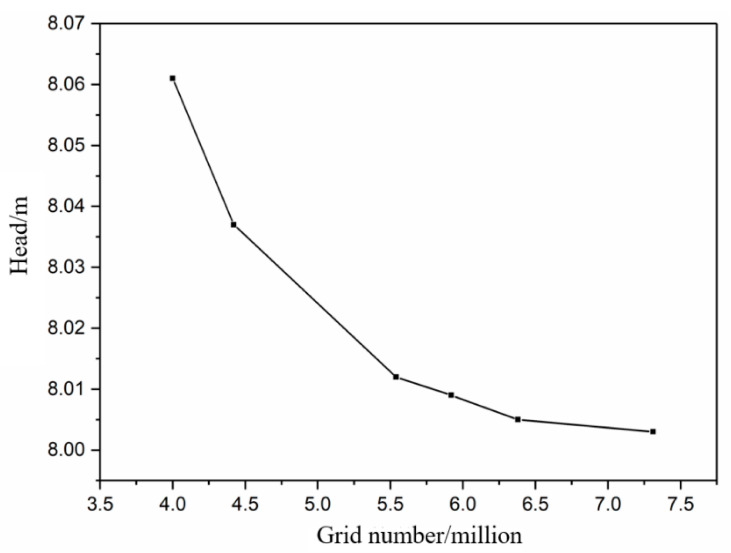
Head of smooth centrifugal pump with different grid number.

Due to the complex boundary of the calculated domain and the huge gap between the bionic scale and the overall size of the blade, it is extremely difficult to divide the structured grid. Therefore, the unstructured grid of the calculated domain was divided by ICEM software. The head of the smooth centrifugal pump with different grid numbers is shown in Figure 4. As can be seen from Figure 4, the change of head tends to be stable when the number of grids reaches about 6.5 million, so the number of grids at this time is sufficient to meet the needs of numerical calculations.

**Figure 5:**
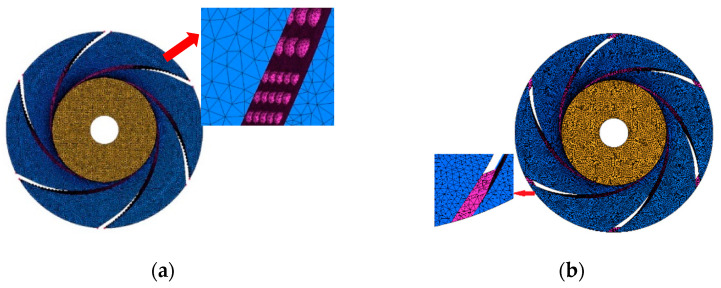
Grid of bionic impeller. (**a**) Bionic pit impeller; (**b**) bionic sawtooth impeller.

The grid of the bionic region was locally refined to ensure that a certain number of nodes were arranged in the bionic position to capture the basic flow structure. At the same time, the size of other grids should be appropriately increased so that the total number of grids does not exceed the computing capacity. The quality of the grid of the bionic impeller is higher than 0.3, and the other calculated domains are higher than 0.35. The physical size of the minimum grid unit is within 0.1mm, and the number of grids reaches about 7.2 million, which ensures that the bionic position has enough fine grids. The grid of the bionic impeller is shown in Figure 5.

### 2.4. Boundary Condition Setting

Ansys CFX 14.5 software was used to calculate the flow field. According to the study of Cravero [16], the *k-ε* turbulence model was selected for the calculation of the flow field. The inlet boundary condition was set at 1 atm, and the outlet boundary condition was set at 4.17 kg/s, 5.56 kg/s, 6.94 kg/s, 8.33 kg/s, 9.72 kg/s, 11.11 kg/s, 12.50 kg/s, 13.89 kg/s, 15.28 kg/s, and 16.67 kg/s flow, respectively. The multiple coordinate systems were used for numerical calculation, the impeller was set as the rotation domain, and the rest of the water body was set as the static domain. The dynamic and static interface was used between the dynamic and static parts, and the static interface was used between the static parts. The fluid medium is 25° clear water, and the calculated reference pressure is at 0 atm. The rotational speed of the impeller is 1450 r/min. The time step of unsteady calculation is 2.29885 × 10^−4^ s, and the total time is 0.57931 s. The convergence criterion was set as the RMS (root mean square) average value, and the convergence accuracy was set at 10^−4^.

The internal sound field was calculated by the direct boundary element method (DBEM) based on LMS Virtual.Lab 13.6 software. Acceleration was taken as the boundary condition, the inlet and outlet were defined as the total sound absorption property, and the rest of surfaces were assumed to be the total reflective wall. Characteristic acoustic impedance *Z* = *ρc* = 1.5 × 106 kg/(m^2^·s), where the velocity of sound *c* = 1500 m/s. The field point was set at 8 times the pipe diameter from the pump outlet flange.

## 3. Verification of Numerical Calculation Method

**Figure 6:**
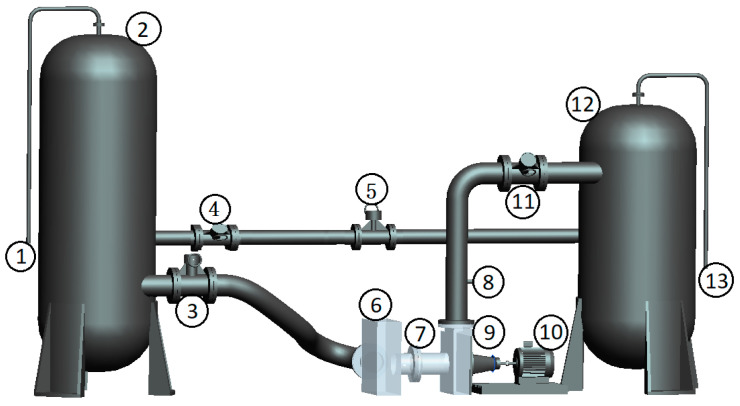
Schematic diagram of closed test platform. 1. Vacuum pump; 2. Cavitation tank; 3, 4, 11. Valve; 5. Electromagnetic Flowmeter; 6. Transparent water tank; 7, 8. Pressure transducer; 9. Test pump; 10. Motor; 12. Voltage-stabilizing tank; 13. Exhaust port.

Bagheri [38] validated the performance of a foil journal bearing solved by the differential quadrature method through tests. A closed pump test platform was built to verify the reliability of the numerical calculation method. The test equipment mainly includes test pump, cavitation tank, vacuum pump, inlet and outlet pipe, valve, electromagnetic flowmeter, motor and pressure transmitter, etc. The test platform is shown in Figure 6.

### 3.1. Flow Field Verification

**Figure 7:**
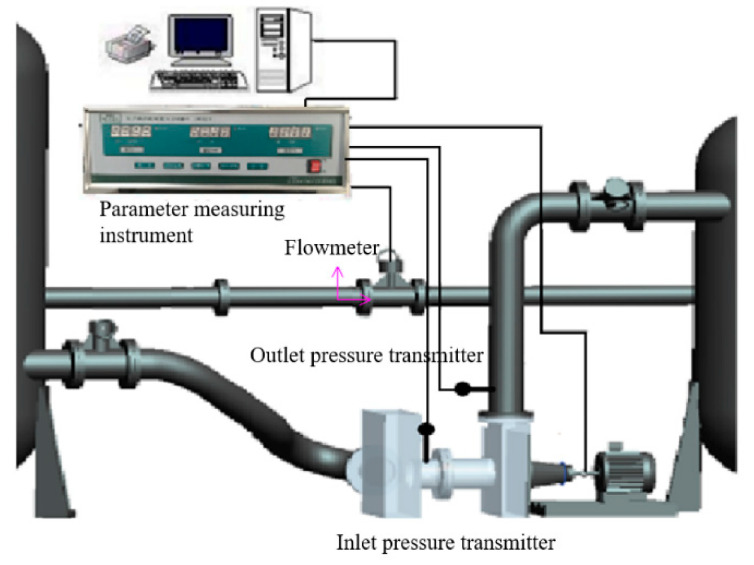
Schematic diagram of intelligent test system for pump parameters.

The external characteristic test of the centrifugal pump was carried out to verify the reliability of the internal flow field calculation. The external characteristic acquisition system is shown in Figure 7. The flowmeter (KEF-DN100 electromagnetic flowmeter) was used to measure the flow of water. The maximum flow calculated with this instrument is 100 m^3^/h, and the accuracy level is 0.5. The model of the pressure transmitter was MIK-P300. The measuring range of the sensor was −0.1~0.1 MPa and 0~1 MPa for the inlet and outlet. Nasiri et al. [39] introduced the type of sensor. The output signal was 4~20 mA current signal, and the accuracy level was 0.5. The measuring point was placed at 2 times the diameter of the inlet and outlet flange.

**Figure 8:**
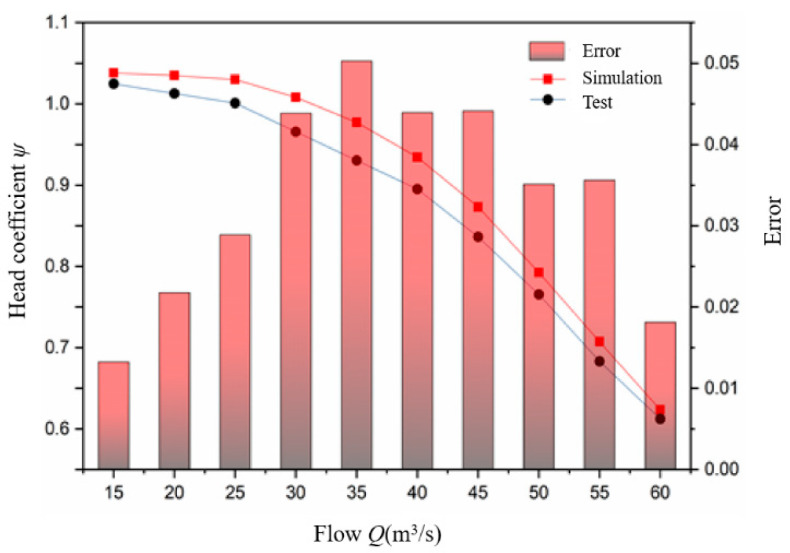
Comparison between test value and simulated value of head coefficient.

The comparison between the test and simulated values of the dimensionless parameter (head coefficient *ψ*) at full flow is shown in Figure 8. The calculation method of *ψ* is shown in Formula (1).

(1) $$\psi =\frac{2gH}{u_{2}^{2}}$$

where *H* is head, *µ*_2_ is the outlet velocity of the impeller, *g* is the acceleration of gravity.

As can be seen from Figure 8, the relative error between the simulated value and the test value is about 5.0%, when the flow is 35 m^3^/h. The relative error is about 4.4%, under the design flow *Q* = 40 m^3^/h. The error of the head coefficient is less than 5% and the simulated value of the head coefficient is consistent with the test value under the full flow, so the numerical calculation method of the flow field has high accuracy.

### 3.2. Sound Field Verification

In order to verify the reliability of the internal sound field calculation, the internal noise test was carried out through the above test system. A high-performance 24-bit sampling instrument (INV3020) was used in the test. The sampling frequency was 12.8 kHz and the sampling time was 30 s. The software DASP V10 was used for data acquisition and signal processing. The internal sound field was tested by an RHSA-10 hydrophone, and the hydrophone was placed 8 times the pipe diameter from the outlet flange by the flush installation method.

Assuming that the medium is an ideal fluid and the propagation process has no energy loss and is an adiabatic process, the acoustic governing equation is used, shown in Formula (2).

(2) $$\frac{{ə}^{2}p}{əx^{2}}+\frac{{ə}^{2}p}{əy^{2}}+\frac{{ə}^{2}p}{əz^{2}}=\frac{1}{c^{2}}\frac{{ə}^{2}p}{ət^{2}}$$

where *p* is the sound pressure, *t* is the time, *c* is the velocity of sound.

**Figure 9:**
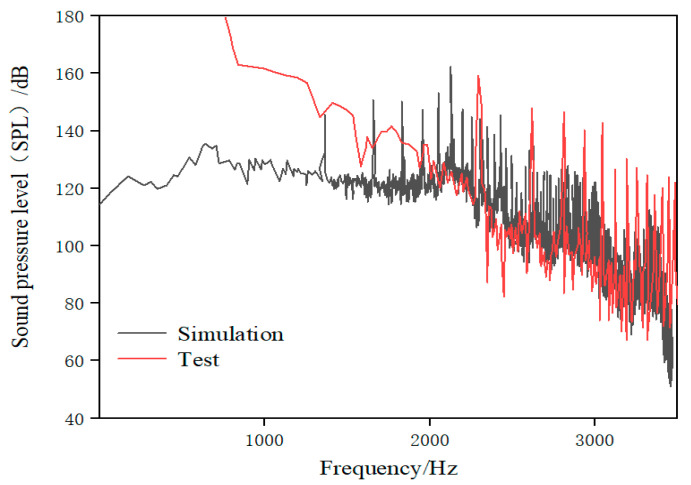
Sound pressure level under rated operating conditions.

The sound pressure level (SPL) obtained by numerical calculation is compared with the test value. The frequency response curve of the sound pressure level under rated operating conditions is shown in Figure 9. The calculation method of the SPL is shown in Formula (3).

(3) $$SPL=20lg\frac{p}{p_{0}}$$

where *p* is sound pressure, *p*_0_ is reference sound pressure, and it is 2 × 10^−6^ Pa in the fluid.

As can be seen from Figure 9, the sound pressure level of the test and simulation is the result of the superposition of discrete and wide-band noise, and there are observable discrete peaks at the blade-passing frequency (BPF) and its frequency doubling. The small sampling frequency of the test results in a large number of peaks on the test spectrum curve. The discrete sound pressure calculated by numerical calculation is higher than in the test, while the wide-band sound pressure is lower, which may because the scattering phenomenon in the inner wall of the pipe is ignored in the calculation. The sound pressure level is close to 149 dB at the axial-passing frequency (24.17 Hz). The sound pressure level values of both test and simulation show a downward trend with the increase in frequency in the frequency range of 100–1000 Hz. The discrete sound pressure on the BPF and its frequency doubling are basically the same between the test and the simulation in the middle- and low-frequency bands. The wide-band sound pressure of the simulation result is generally lower than in the test after the BPF. However, the difference is about 10 dB, so it can still reflect the actual changing trend of noise. The wide-band sound pressure level of simulation is consistent with the test in the range of 1000–2000 Hz, and both fluctuate slightly in the range of 80–90 dB. The discrete peaks of the test results are lower than the simulated ones at the high-order BPF, which is the same as that described by Liu et al. [40]. Moreover, there is a certain difference in sound pressure level between the test and simulation, as they both decrease in a step-wise manner and have similar discrete noise amplitude within a certain frequency range.

The numerical calculation results are inevitably different from the test in the specific sound pressure level values, but they can show most of the laws of the amplitude–frequency characteristics of the actual noise. Therefore, the numerical calculation method of the internal sound field has a certain reference value.

## 4. Comparison of Bionic Pit and Bionic Sawtooth

In this section, the effects of pit and sawtooth on the performance of the centrifugal pump are studied.

### 4.1. Comparison of Performance Parameters

The hydraulic performance parameters, acoustic performance parameters, and change rate of the basic model and two different bionic models under design conditions are shown in Table 2 and Table 3.

It can be seen from Table 2 that the pit structure has little influence on the external characteristic parameters of the basic model. The change rate of the head is 0.9%, torque is 1.3%, and hydraulic efficiency is −0.3%. This is mainly due to the fact that the pit structure has little effect on the length and width of the flow passage. However, the sawtooth structure reduces the radius of the impeller and thus reduces the head. The sawtooth structure reduces the head of the basic model by 9.4%, torque by 8.9%, and hydraulic efficiency by 0.5%. In accordance with the conclusion, the sawtooth structure has great influence on the performance of the centrifugal pump, while the pit structure has little influence. However, both structures have a certain noise reduction effect, and the noise reduction effect of the sawtooth structure on the total sound pressure level is 3.94 dB, which is 3.07 dB higher than the pit structure. However, in terms of wide-band total sound pressure level, the pit structure has a noise reduction of 0.94 dB, which is 0.65 dB higher than the sawtooth structure. The sawtooth structure is likely to have a good suppression effect on the discrete noise with high amplitude, so as to reduce the total sound pressure level, but it is difficult to reduce the wide-band noise.

The pit structure makes the hydraulic efficiency of the centrifugal pump decrease slightly, but it is improved in the relevant references. There are three possible reasons. Firstly, the model is a twisted blade, and its working mechanism is likely to be contrary to the bionic structure. Secondly, the model pump used in this paper has higher efficiency as compared with the previous related studies [18,41,42]. The results also stated that, if one of them (increasing efficiency and noise reduction) approaches the maximum value, this leads to the decline of the other parameter. In addition, the non-smooth structure makes the optimum operating point of the centrifugal pump deviate, in accordance with similar results [41]. Therefore, the efficiency of bionic centrifugal pumps may also be improved for all operating conditions.

### 4.2. Comparison of Acoustic Characteristics

**Figure 10:**
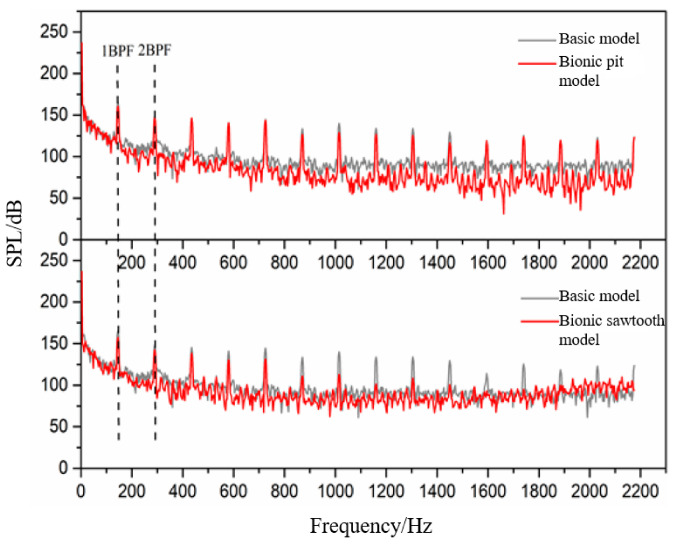
Frequency domain diagram of sound pressure level.

Figure 10 shows the frequency domain diagram of the sound pressure level of the basic model and two different bionic models. It can be seen that there is an observable difference between pit and sawtooth structures for noise reduction. The noise reduction ability of the pit structure is mainly due to the suppression of wide-band noise. The noise reduction ability of the sawtooth structure involves restraining the discrete noise at the BPF and its frequency doubling. Therefore, it has a relatively great influence on the total sound pressure level.

The bionic pit model can reduce wide-band noise at almost all frequencies, especially after 7 BPF. The sawtooth model struggles to achieve noise reduction in the wide-band. The sawtooth model has a higher sound pressure level than the basic model from 1600 Hz, which is consistent with the test results of Gruber [23]. He suggested that it may be caused by the microjet at the root of the sawtooth. Compared with the sawtooth structure, the noise reduction ability of the pit structure is more suitable for reducing the wide-band noise in the high-frequency band. The statistical results of the wide-band concluded that the total sound pressure level within 7–14 BPF of the basic model is 177.53 dB, that of the bionic sawtooth model is 177.46 dB, and that of the bionic pit model is 175.97 dB. However, the pit structure is limited by its acting area in the flow field and has little effect on noise reduction at the BPF, which can effectively reduce the wide-band noise in the whole frequency band.

**Figure 11:**
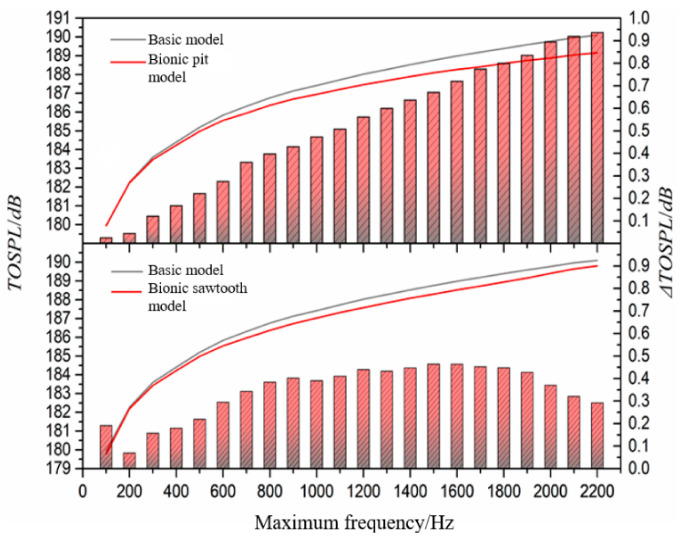
Relationship between noise reduction value and calculation frequency.

In order to further explore the wide-band noise reduction ability of the bionic structure, the statistical maximum frequency is changed, and the wide-band total sound pressure level from the lowest frequency to the highest frequency is calculated. The statistical results are shown in Figure 11. The results stated that the gap between the curve of the basic model and the bionic pit model is widened, but the curves of the basic model and the bionic sawtooth model tend to intersect. The noise reduction effect of the bionic pit structure on the wide-band total sound pressure level increases with the increase in the maximum calculation frequency. However, the weakness of the wide-band noise suppression ability of the sawtooth structure is gradually reflected in the high-frequency band above 1200 Hz. The difference of the wide-band noise between the bionic sawtooth model and the basic model continues to narrow from 1600 Hz, indicating that the sawtooth structure increases the wide-band noise at high frequency.

**Figure 12:**
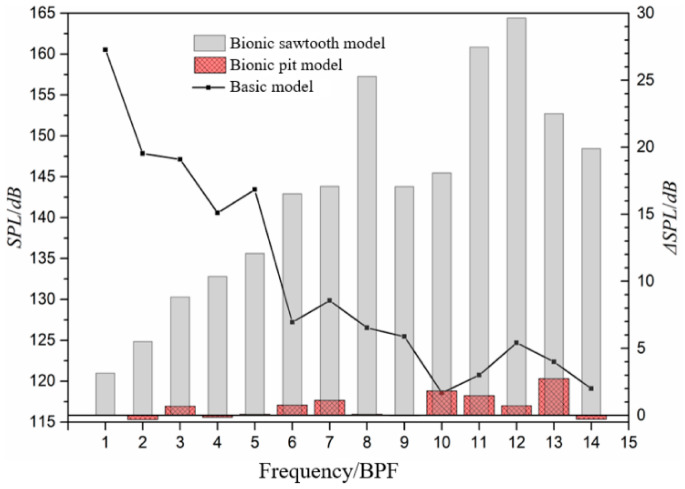
Sound pressure level at blade-passing frequency (BPF) and noise reduction value of bionic structure.

The influence of different bionic structures on discrete sound pressure level is shown in Figure 12. With the increase in the frequency, the sound pressure level at the BPF shows a downward trend, while the noise reduction value shows an upward trend. The noise reduction value of the bionic sawtooth model in the BPF can reach about 30 dB, which is much higher than the bionic pit model. In terms of the mechanism of fluid noise, the pit structure arranged on the blade surface mainly affects the fluid near the boundary layer, and it is difficult to improve the discrete noise caused by the interference between the blade and the volute tongue. Even the noise at the first two discrete frequencies is increased by the pit structure.

### 4.3. Analysis of Internal Flow Field

The strong three-dimensional flow process in the twisted blade significantly affects the flow principle of the bionic structure, and affects the noise reduction mechanism. Therefore, this section focuses on the influence of the bionic structure on the internal flow field.

**Figure 13:**
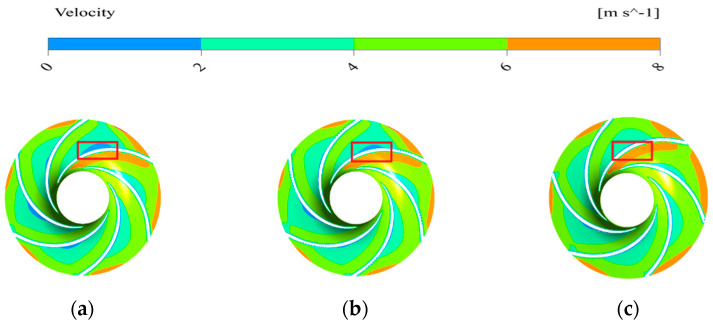
Velocity distribution on the middle section of different models. (**a**) Basic model; (**b**) bionic pit model; (**c**) bionic sawtooth model.

The velocity distribution on the middle section is shown in Figure 13. It can be seen from the Figure 13 that there is a low-velocity fluid mass on the pressure surface of the impeller passage, and the volume of the low-velocity fluid mass is the largest in the basic model, but almost negligible in the bionic sawtooth model. The fluid velocity on the suction surface of the impeller is relatively stable, which is determined by the structure and the working mode of the impeller.

**Figure 14:**
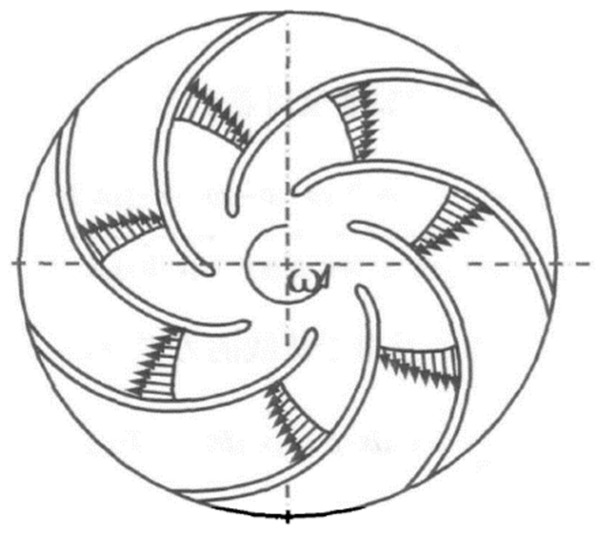
Distribution of relative velocity in impeller.

Figure 14 shows the variation of the relative velocity of the impeller on the section parallel to the axis. The change of relative velocity on the pressure surface is not equal to the suction surface. The fluid usually has a large velocity gradient on the second half of the pressure surface, and the middle part of the pressure surface usually becomes the accumulation area of low-velocity fluid. The blade load depends on the difference between the relative velocity of the pressure surface and the suction surface at the same radius of the blade. The reduction of the volume of low-velocity fluid by increasing the bionic structure is beneficial to uniform the load of blade and to avoid the phenomenon of flow separation caused by small fluid velocity near the pressure surface.

**Figure 15:**
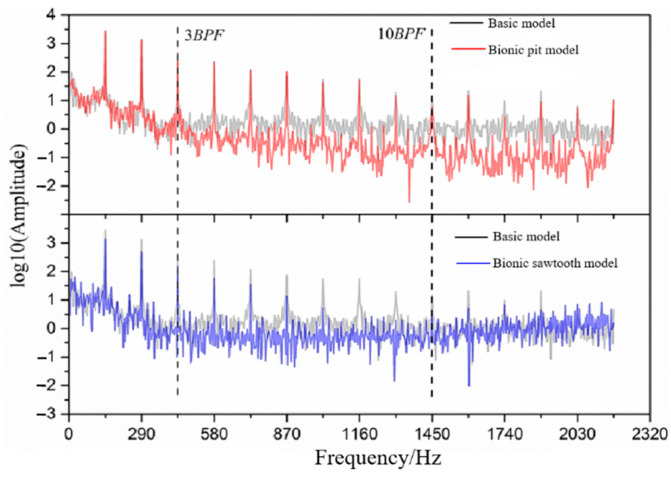
Frequency domain diagram of pressure fluctuation.

The frequency domain diagram of pressure fluctuation of different models near the volute tongue is obtained by Fourier transform [43], as shown in Figure 15. The difference between the extreme and mean value of the original data of pressure fluctuation is large, so the logarithmic function with base 10 is used to deal with the amplitude data of pressure fluctuation. As can be seen from Figure 15, the pressure pulsation has an obvious peak at the BPF, which is similar to the sound pressure level. The sawtooth structure can significantly reduce the amplitude of pressure fluctuation at the BPF and its frequency doubling. The sawtooth structure can basically reduce the amplitude of pressure fluctuation in the frequency band between 3 BPF and 10 BPF. The pit structure begins to show a good ability to reduce the amplitude of wide-band pressure fluctuation from 3 BPF. The amplitude of pressure fluctuation near the volute tongue approximately reflects the amplitude–frequency characteristic of sound pressure.

The noise produced by the impeller can be divided into two types: one is caused by the interference between the boundary layer and the trailing edge, and the other is caused by the dynamic and static interference between the impeller and the volute tongue. Noise reduction mechanisms of pit and sawtooth structures are obviously different, corresponding to the first and second types mentioned above, respectively. There are unstable disturbance factors near the wall or boundary layer in the impeller passage, such as Tollmien–Schlichting waves. These unstable disturbances transfer along the boundary layer to the trailing edge and eventually interfere with the trailing edge, resulting in noise. However, there is a special vortex structure in the pit or the pit itself interferes with or absorbs the unstable disturbance. The filtering effect on unstable factors may depend on the relative size of the pit or special vortex structure and the wavelength of the unstable disturbance. In addition, according to the acoustic feedback loop proposed by Tam [44], Fink [45], and Arbey [46], the noise near the outlet of the impeller is the result of the superposition of different sound waves. The sound waves generated by the disturbance in the boundary layer and trailing edge interference partially radiate upstream, which is superimposed with the unstable disturbance waves in the upstream boundary layer. The superimposed disturbance wave produces corresponding feedback according to the phase difference. If the phase is the same, the disturbance wave is amplified, and the amplified disturbance wave reaches the trailing edge and radiates again to produce larger sound waves. After the sound wave is superimposed, it radiates to the upstream again, and finally forms a feedback loop. The difference in the diameter of the pit makes the phase of the nearby unstable disturbance wave different, and then affects the phase difference between the disturbance wave and the trailing edge noise feedback sound wave and the superposition effect.

## 5. Conclusions

Pit and sawtooth bionic tests were carried out on the blade to explore the influence of different bionic structures on the noise reduction performance of the centrifugal pump. The hydraulic performance, internal flow field, and internal sound field of the centrifugal pump were calculated by the numerical simulation method. The conclusion of this research can be summarized as follows:

(1) There is an obvious difference in the external characteristic parameters between the bionic pit and the bionic sawtooth model. The pit structure has little influence on the external characteristic parameters of the basic model, while the sawtooth structure has a great influence on the external characteristic parameters.
(2) The noise decrement effect of the pit structure is apparently different from that of the sawtooth structure. The noise reduction effect of the pit structure is aimed at the wide-band noise, and the sawtooth structure is aimed at the discrete noise at the BPF and its frequency doubling. The highest noise reduction value of the sawtooth structure is noted to be about 30 dB at 12 BPF. The change of discrete noise has a significant effect on the total sound pressure level. When the total sound pressure level is taken as the index, the noise reduction of the sawtooth structure is 3.94 dB, which is 3.13 dB higher than that of the pit structure. However, when the wide-band total sound pressure level is taken as the index, the noise reduction of the pit structure is 0.94 dB, which is 0.65 dB higher than that of the sawtooth structure.
(3) The wide-band noise reduction ability of the sawtooth structure is not suitable for the high-frequency band. On the frequencies other than the BPF and its frequency doubling, the sawtooth structure almost loses its noise reduction ability from 1200 Hz. The sawtooth structure causes the noise to increase when the frequency exceeds 1600 Hz. The results of pressure fluctuation near the volute tongue also show that the sawtooth structure causes the amplitude of pressure fluctuation after 1600Hz to exceed the basic model.

The research in this paper is of great significance to reducing the noise of centrifugal pumps, whether it is wide-band noise or discrete noise. It can be a reference for the noise reduction of centrifugal pumps. There is still some work to be studied in depth, such as the best size of bionic structures.

## Figures and Tables

**Table 1:** Main geometric parameters.

Overcurrent Component	Geometrical Parameter	Symbol	Numerical Value
Impeller	Inlet diameter (mm)	*D*_1_	90
	Outlet diameter (mm)	*D*_2_	170
	Exit width (mm)	*b*_2_	13.1
	Blade wrapping angle (°)	*φ*	120
	Blade number	*z*	6
Volute	Base circle diameter (mm)	*D*_3_	180
	Inlet width (mm)	*b*_3_	32
	Outlet diameter (mm)	*D_d_*	80

**Table 2:** External characteristic parameters of different models under design conditions.

Model	Head (m)	Change Rate (%)	Torque (N·m)	Change Rate (%)	Hydraulic Efficiency (%)	Change Rate (%)
Basic model	7.94	-	7.18	-	0.793	/
Bionic pit	8.01	0.9	7.27	1.3	0.791	−0.3
Bionic sawtooth	7.19	9.4	6.54	−8.9	0.789	−0.5

**Table 3:** Total sound pressure level (TOSPL) and wide-band total sound pressure level of different models under design conditions.

Model	Total Sound Pressure Level (dB)	Noise Reduction Value (dB)	Wide-Band Total Sound Pressure Level (dB)	Noise Reduction Value (dB)
Basic model	166.95	-	190.10	-
Bionic pit	166.14	0.81	189.16	0.94
Bionic sawtooth	163.01	3.94	189.81	0.29

## Data Availability

The data presented in this study are available in this published article.
